# Acupuncture for ulcerative colitis: a systematic review and meta-analysis of randomized clinical trials

**DOI:** 10.1186/s12906-020-03101-4

**Published:** 2020-10-14

**Authors:** Xiao Wang, Nan-qi Zhao, Yu-xin Sun, Xue Bai, Jiang-tao Si, Jian-ping Liu, Zhao-lan Liu

**Affiliations:** 1 Center for Evidence-Based Chinese Medicine, Beijing University of Chinese Medicine, Beijing, 100029 China; 2 Special Treatment Center, Wang Jing Hospital of China Academy of Chinese Medicine Sciences, 100102 Beijing, China

**Keywords:** Acupuncture, Ulcerative colitis, Systematic review, Meta-analysis, Randomized controlled trials, Clinical evidence

## Abstract

**Background:**

Ulcerative colitis, characterized by diarrhea, bloody stools and abdominal pain, is a chronic, idiopathic inflammatory disease of the colonic mucosa. In recent years, the incidence of ulcerative colitis presents an increasing trend year by year. Acupuncture, as a potential effective treatment for ulcerative colitis, is widely used in clinical practice.

**Methods:**

We searched PubMed, the Cochrane Library, Chinese CBM Database, China National Knowledge Infrastructure, Chinese VIP Information, and Wanfang Database from the date of the establishment of each database up to March, 2019. We included randomized controlled clinical trials (RCT) comparing acupuncture versus conventional conventional medicine or comparing acupuncture combined with conventional medicine versus conventional medicine in participants with ulcerative colitis. Two authors screened all references, assessed the risk of bias and extracted data independently. We summarized data using risk ratios (RR) with 95% confidence intervals (CI) for binary outcomes. We performed meta-analyses using random effects model. We assessed overall quality of evidence using GRADE.

**Results:**

We included 13 RCTs (1030 participants, 515 in the acupuncture group and 515 in the control group). Only one study tested head acupuncture, and the other 12 tested body acupuncture. The treatment duration ranged from 14 to 60 days. Seven trials compared acupuncture alone versus conventional medicine, and six compared acupuncture combined with conventional medicine versus conventional medicine. Acupuncture combined with mesalazine showed better clinical effect (improved clinical symptoms, colonoscopy results and stool examination results) (RR 1.25, 95% CI 1.19 to 1.41; 232 participants; 4 trials; low quality evidence) and better colonoscopy curative effect (RR 1.33, 95% CI 1.04 to 1.71; 108 participants; 2 trials; moderate quality evidence) compared to mesalazine. Acupuncture showed better clinical effect compared to the combination of metronidazole and sulfasalazine (RR 1.21, 95%CI 1.10, 1.34; 318 participants; 3 trials; moderate quality evidence). There was no significant difference in the incidence of adverse events between groups.

**Conclusions:**

Both acupuncture alone and acupuncture combined with conventional medicine may be effective in treating ulcerative colitis compared to conventional medicine. Our findings must be interpreted with caution due to high or unclear risk of bias of the included trials.

## Background

Ulcerative colitis (UC) is a chronic idiopathic inflammatory disease of the colonic mucosa, manifested as persistent or recurrent diarrhea, mucous purulent blood stool, abdominal pain and systemic symptoms of varying degrees, for more than 4–6 weeks. The lesions may involve the rectum, sigmoid colon, left colon, right colon and total colon [1]. Except for involvement of parts of gastrointestinal tract, extraintestinal manifestations may occur involving skin, mucosa, joints, eyes, liver and gallbladder [2]. The severity of UC can be divided into mild, moderate and severe grades [1]. It most often occurs in young adults. As estimated in China, the peak age of onset for UC is 20–49 years old, with no significant difference between male and female [3, 4]. The prevalence of UC in China is about 11.6/100,000 [5]. In recent years, with the improvement of people’s living standard along with the continuous progress of detection technology, the incidence of UC presents an increasing trend year by year. The prevalence of UC in southeast Asian countries including China has doubled during the past 10 years. Moreover, due to long disease course and its frequent recurrence, the quality of life (QOL) of UC patients has been seriously affected [3, 6].

Acupuncture is often used in the clinical practice of treating UC patients. A systematic review published in 2016 compared the effectiveness of acupuncture to sulfasalazine in the treatment of UC; however, the review also included trials of acupuncture in combination with moxibustion and did not report the results separately, which deterred us from knowing the effects of using acupuncture only for UC. Moreover, the difference between acupuncture and other conventional medicine instead of sulfasalazine was unclear [7]. Another previously published systematic review explored the effects of acupuncture for treating UC, but it has been published in 2007 and has to be updated [8]. With the increasing number of randomized clinical trials (RCTs) on this topic getting published in recent years, it is necessary to update the evidence. Therefore, we conducted this study to explore the effectiveness of acupuncture for UC, so as to provide reference for clinical practice.

## Methods

The protocol of the review was registered in PROSPERO on 13th April 2019 (Registration number: CRD42019132172; available from: http://www.crd.york.ac.uk/PROSPERO/). The review was conducted and reported according to the Preferred Reporting Items for Systematic Reviews and Meta-Analyses (PRISMA) [9].

**Inclusion criteria**

1. Types of studies: only RCTs were included.
2. Types of participants: we included participants aged 18 years or older given the diagnosis of ulcerative colitis defined by clear diagnostic criteria or references. There was no limitation of gender, course of disease, and severity.
3. Types of interventions: the intervention should be acupuncture or acupuncture in combination with conventional conventional medicine. The original literature needs to have a clear description of acupuncture process, such as disinfection and sterilization, acupuncture manipulation, post-treatment process.
4. Types of controls: control measures should be conventional conventional medicine, with clear reporting of the method of medication, dosage and course of treatment.
5. Types of outcome measures: primary outcomes were clinical effect (the improvement of clinical symptoms, colonoscopy results and stool examination results) and colonoscopy curative effect; secondary outcome was adverse reactions.

### Study identification and selection

We searched PubMed, the Cochrane Library, Chinese CBM Database, China National Knowledge Infrastructure (CNKI), Chinese Scientific Journal Database (VIP), and Wanfang Database from the date of the establishment of each database up to March, 2019. Search terms included “ulcerative colitis”, “UC”, “acupuncture”, and “random”.

Two authors in pairs (X Wang and NQ Zhao) screened the titles and abstracts of articles in NoteExpress independently. After screening abstracts, full text of the articles were downloaded and read. In case of disagreement between the two authors, the third author shall arbitrate (YX Sun).

### Data extraction and quality assessment

We extracted the data into Microsoft Excel 2010 and collected the following information: (1) basic information of included studies: ID (Author’s initials + year), publication language, publication year, sample size, intervention measures, control measures and treatment course; (2) patient information: age, gender, course of disease, severity and disease stage; (3) outcome measurements: primary outcomes: clinical effect, colonoscopy curative effect; secondary outcome: adverse reactions.

Two review authors in pairs (NQ Zhao and YX Sun) independently used the Cochrane Risk of bias assessment tool to determine the risk of bias for each included trial [10]. We resolved any disagreements by consensus or by consulting a third review author (X Wang). Risk ratings of “low”, “high” or “unclear” were assigned to the following items: random sequence generation, allocation concealment, blinding of participants and personnel, blinding of outcome assessment, incomplete outcome data, selective reporting and other biases.

### Data analysis

We used RevMan 5.3 software for data analysis. For clinical effect, colonoscopy curative effect and adverse events, we presented as relative risk (RR) with 95% confidence interval (CI) (after-intervention values were used to calculate the effect estimate). Statistical analysis was performed according to the statistical guidelines cited in the latest Cochrane Handbook for Systematic Reviews of Interventions [10]. We performed meta-analyses if the trials had good homogeneity on study design, participants, intervention, control, and outcomes. We performed meta-analyses using random effects model. The *I*^2^ statistic was used to calculate statistical heterogeneity. If the heterogeneity between studies was significant (*I*^2^>75%), we would not perform meta-analysis and the source of heterogeneity should be analyzed. We performed subgroup analysis where different types of controls were used. When more than 10 RCTs were available to test the same outcome in one meta-analysis, we used funnel plots to intuitively assess publication bias. We used GRADE to assess the overall quality of evidence [11].

## Results

### Description of the literature

**Fig. 1:**
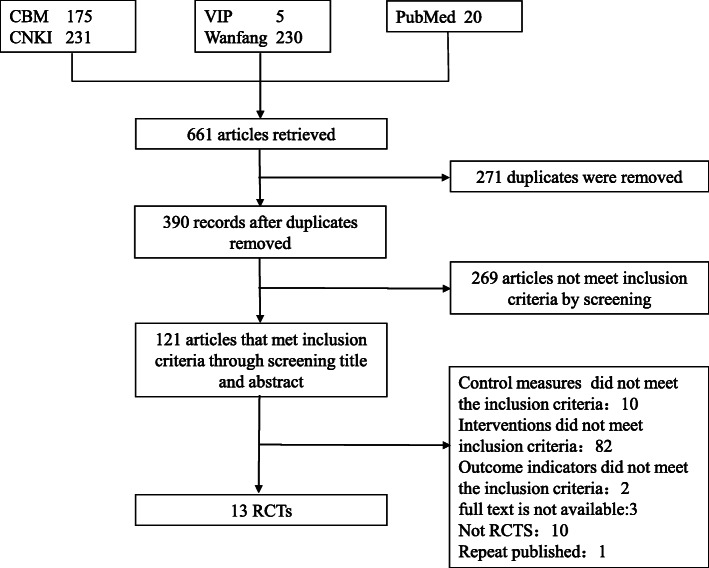
Literature screening flow chart

A total of 661 literatures were retrieved, and 121 remained after screening titles and abstracts. We read the full text of these 121 literatures, and by excluding 108 literatures we finally included 13 literatures [12–24]. The screening process is shown in Fig. 1.

### Basic information of included trials

We included 13 RCTs (1030 participants, 515 in the treatment group and 515 in the control group). All of the trials were conducted in China and were published in Chinese. Seven trials compared acupuncture versus conventional conventional medicine [12, 15, 17, 18, 20, 21, 23], and six compared acupuncture in combination with conventional conventional medicine versus conventional conventional medicine [13, 14, 16, 19, 22, 24]. Only one trial was treated with head acupuncture [12], and the other 12 were treated with body acupuncture. The treatment duration ranged from 14 to 60 days. Participants were aged 20–78 years old. The course of illness was 9 days-8 years. There were 543 males and 487 females. Characteristics of included trials are shown in Table 1.

**Table 1:** Characteristics of included randomized trials of acupuncture for ulcerative colitis

Study ID	Year	Gender male/female	Sample size	Age (mean or range, yrs)	Course of disease (mean or range)	Course of treatment, days	Intervention vs. Control	Outcomes	Acupuncture intervention details					Control measures
									Acupuncture point [25]	Acupuncture on one or both sides of the body	Duration of each treatment	Frequency	With or without conventional medicine	
RenY2014	2014	I:10/14 C:12/12	I:24 C:24	I:46.25 ± 15.11 C:50.2 ± 14.19	I:5.08 ± 2.1 years C:4.83 ± 1.74 years	46	Acupuncture + mesalazine vs. mesalazine	Clinical effect, activity index for UC, TCM syndrome score, colonoscopy curative effect, mucosal pathology, adverse effects	BL23, BL20, BL25, BL21, SP4, ST25, ST36, KI3, CV12, CV4	both sides	30 min	1 time/day	Y	mesalazine: 1 time/day, 1.0 g/time
JiaJN2015	2015	I:16/16 C:18/14	I:32 C:32	I:28–60 C:24–57	I:1–8 years C:1–7 years	60	Acupuncture + mesalazine vs. mesalazine	Clinical effect, T Cell subsets	CV3, CV4, CV6, ST25, SP15, BL25, ST36, ST37, SP6, LR3	NR	30 min	1 time/day	Y	mesalazine: 4 times/day, 1.0 g/time
YangSQ2012	2012	I:14/16 C:15/15	I:30 C:30	I:24–49 C:25–53	I:0.5–5 years C:0.7–6 years	28	Acupuncture + mesalazine vs. mesalazine	Clinical effect, clinical symptom score, colonoscopy curative effect, self-rating depression scale, adverse effects	Selected the frontal area as the therapeutic area by seven - area division of the head acupoints	NR	6 h	NR	Y	mesalazine: 4 times/day, 1.0 g/time
XueLZ2018	2018	I:25/22 C:24/23	I:47 C:47	I:28–75(40.3 ± 13.9) C:29–74(40.1 ± 12.8)	I:NRC:NR	14	Acupuncture vs. metronidazole + sulfasalazine	Clinical effect, adverse effects	CV4, CV6, GV1, BL25, ST25, SP6, ST36	NR	20 min	1 time/day	N	metronidazole: 3 times/day, 0.2 g/time; sulfasalazine: 3 times/day,0.2 g/time
LiuXH2013	2013	I:29/33 C:30/32	I:62 C:62	I:23–76(50.67 ± 6.82) C:24–74(51.14 ± 5.46)	I:9–19(13.63 ± 5.16) months C:9–20(14.10 ± 5.22) months	–	Acupuncture vs. metronidazole + sulfasalazine	Clinical effect, adverse effects	CV4, CV6, ST25, BL25, GV1, ST36, SP6	NR	10 -30 min	2 times/day	N	metronidazole: 3 times/day, 2-3 g/time; sulfasalazine: 3 times/day, The starting dose is 2–3 g/day, which can be increased to 4–6 g/day according to the patient’s condition. After the patient’s condition is stable, the dosage can be reduced to 1.5 to 2 g/day
LiCH2017	2017	I:25/25 C:26/24	I:50 C:50	I:27–75(45.5 ± 4.5) C:27–76(46.8 ± 4.2)	I:9–18(13 ± 1.5) months C:10–18(12 ± 1.8) months	–	Acupuncture vs. metronidazole + sulfasalazine	Clinical effect, pathological change ratio	CV4, CV6, ST25, GV1, BL25, SP6, ST36	NR	10-30 min	2 times/day	N	metronidazole: 3 times/day, 0.2 g/time; sulfasalazine: 3 times/day, 2.0 g/time
ZhangZZ2018	2018	I:29/21 C:27/23	I:50 C:50	I:32–66(44.1 ± 3.2) C:31–65(43.6 ± 3.0)	I:1–9(3.6 ± 1.5) months C:1–8(3.5 ± 1.7) months	14	Acupuncture vs. mesalazine	Clinical effect, IL-2	ST25, ST36), GV1, SP6	NR	16-22 min	1 time/day	N	mesalazine: 3 times/day, 1.0–2.0 g/time
WangMC2017	2017	I:12/13 C:17/8	I:25 C:25	I:38–74(56.8 ± 2.4) C:36–69(52.5 ± 3.8)	I:1 month-4 years C:1 month-3 years	10	Acupuncture vs. mesalazine	Clinical effect, adverse effects	ST25, SP6, GV1, ST36, BL18, BL20, BL23	NR	20 min	1 time/day	N	mesalazine: 3 times/day, 2.0 g/time
LuanBY2016	2016	I:13/12 C:14/11	I:25 C:25	I:26–42(34 ± 5.75) C:23–42(31.28 ± 6.13)	I:NR C:NR	56	Acupuncture vs. mesalazine	Clinical effect, adverse effects	SP4, KI3, ST36, CV4, ST25, BL16, BL20, BL21, BL22, BL25, GV2, GV6	NR	30 min	1 time/day	N	mesalazine: 4 times/day, 1.0 g/time
ZhangHC2009	2009	I:23/17 C:25/15	I:40 C:40	I:20–65(42.64 ± 6.9) C:19–67(38.73 ± 7.5)	I:2.2–7.9(3.9 ± 2.3) years C:2.2–7.4(4.1 ± 1.7) years	28	Acupuncture vs. sulfasalazine	Clinical effect, adverse effects	Gastric region was selected according to functional localization of cerebral cortex	NR	5-10 min	1 time/day	N	sulfasalazine: 3 times/day, 2.0 g/time
YanZL2018	2018	I:25/20 C:23/22	I:50 C:50	I:25–78(42.6 ± 11.7) C:26–77(41.3 ± 12.5)	I:NR C:NR	14	Acupuncture + metronidazole + sulfasalazine vs. metronidazole + sulfasalazine	Clinical effect	ST25, BL25, GV1, CV6, CV4, ST36, SP6	NR	20 min	1 time/day	Y	metronidazole: 3 times/day,0.2 g/time; sulfasalazine: 3 times/day, 0.2 g/time
ZhangCY2018	2018	I:30/20 C:26/24	I:50 C:50	I:34–70(46.5 ± 0.5) C:35–69(45.6 ± 0.01)	I:9 days-2 years (1.2 ± 0.01) years C:10 days-3 years (2.1 ± 0.01) years	30	Acupuncture + metronidazole vs. metronidazole	Clinical effect, adverse effects	CV4, CV6, ST25, GV1, BL25, SP6, ST36	NR	10-30 min	2 times/day	Y	metronidazole: 3 times/day,0.2 g/time
PangHM2020	2020	I:16/14 C:19/11	I:30 C:30	I: 41.63 ± 12.86 C: 43.33 ± 15.51	I: 36.90 ± 20.94 months C: 38.03 ± 18.42 months	30	Acupuncture + mesalazine vs. mesalazine	Clinical effect, Baron score	BL31, BL32, BL33,BL34	both sides	30 min	1time/2 days	Y	mesalazine: 4 times/day, 1.0 g/time

### Risk of bias in included trials

**Fig. 2:**
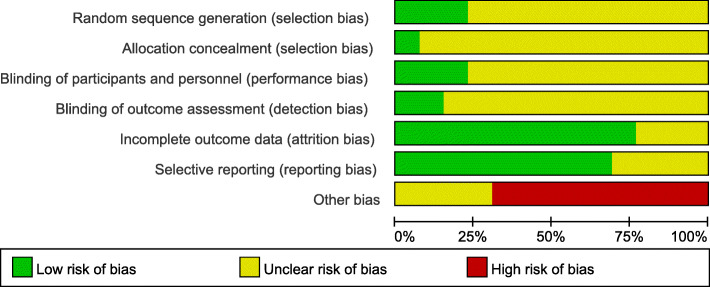
Risk of bias of randomized clinical trials of acupuncture for ulcerative colitis

A total of three studies reported the method to randomize participants, by using random number table or dynamic block randomization, which were considered as low risk of bias [16, 19, 24]. Only one study reported adequate allocation concealment, which was considered as low risk of bias [16].None of the studies reported whether participants and outcome assessors were blinded, which were considered unclear risk of bias. In terms of other bias, eight studies which had only one author were considered to have a high risk of bias when the whole trial was finished by one author [13, 17–21, 23, 24]. The details of bias of risk assessment of included studies is shown in Fig. 2. Additionally, the risk of bias of each study incorporated with forest maps was described using a dot plot. A: random sequence generation (selection bias); B: allocation concealment (selection bias); C: blinding of participants and personnel (performance bias); D: blinding of outcome assessment (detection bias); E: incomplete outcome data (attrition bias); F: selective reporting (reporting bias); G: other bias.

### Primary outcomes

#### Clinical effect

The standard of clinical effect refers to the *Consensus on the diagnosis and treatment of inflammatory bowel disease* [2]. Clinical symptoms and endoscopic examination were used as the criteria for effectiveness evaluation. Clinical effect was reported in all studies.

#### Acupuncture versus conventional conventional medicine

**Fig. 3:**
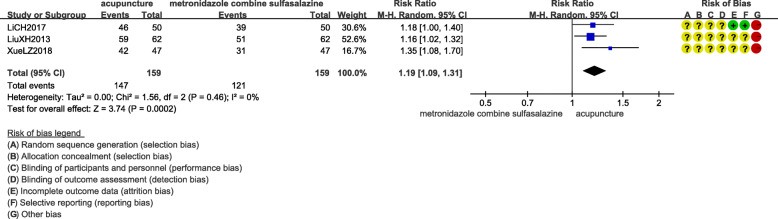
Clinical effect of acupuncture alone versus metronidazole combined with sulfasalazine

Acupuncture alone was 1.19 times more effective than metronidazole combined with sulfasalazine (RR 1.19, 95%CI 1.09, 1.31; 318 participants; 3 trials; moderate quality evidence) (Fig. 3) [18, 20, 22]. There was no significant difference between acupuncture and mesalazine on clinical effect (RR 1.05, 95%CI 0.80 to 1.37; 200 participants; 3 trials; very low quality evidence) [15, 17, 23]. One RCT compared the clinical effect of acupuncture versus sulfasalazine, and the result showed that there was no statistical difference between two groups (RR 0.67, 95%CI 0.28 to 1.62; 80 participants; 1 trial; low quality evidence) [12].

#### Acupuncture combined with conventional conventional medicine versus conventional conventional medicine

**Fig. 4:**
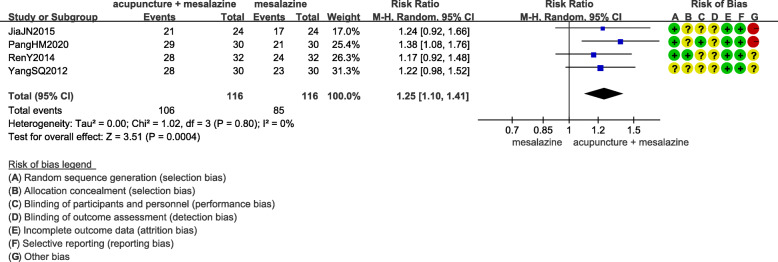
Clinical effect of acupuncture combined with mesalazine versus mesalazine alone

The clinical effect of acupuncture combined with mesalazine was 1.25 times more than that of mesalazine alone (RR 1.25, 95%CI 1.19 to 1.41; 232 participants; 4 trials; low quality evidence)(Fig. 4) [14, 16, 19, 24]. One RCT compared acupuncture combined with metronidazole and sulfasalazine versus metronidazole combined with sulfasalazine, and the result showed that the addition of acupuncture group had better clinical effect (RR 1.24, 95%CI 1.04 to 1.47; 100 participants; 1 trial; low quality evidence) [21]. One RCT compared acupuncture combined with metronidazole versus metronidazole alone, and the result showed that acupuncture group had better clinical effect (RR 1.29, 95%CI 1.05 to 1.58; 100 participants; 1 trial; low quality evidence) [13].

#### Colonoscopy curative effect

**Fig. 5:**
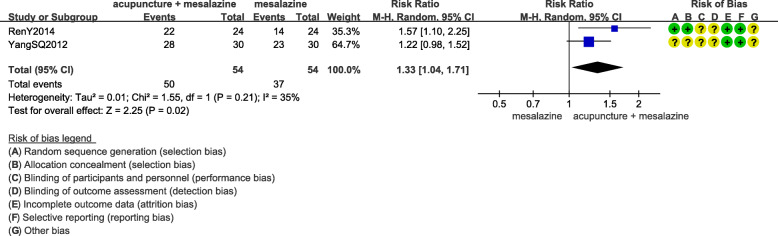
Colonoscopy curative effect of acupuncture combined with mesalazine versus mesalazine alone

Two RCTs reported colonoscopy curative effect [14, 16]. The meta analysis showed that the colonoscopy curative effect of acupuncture combined with mesalazine was 1.35 times higher than that of mesalazine alone (RR 1.33, 95%CI 1.04 to 1.71; 108 participants; 2 trials; moderate quality evidence) (Fig. 5).

### Secondary outcomes

#### Adverse events

**Fig. 6:**
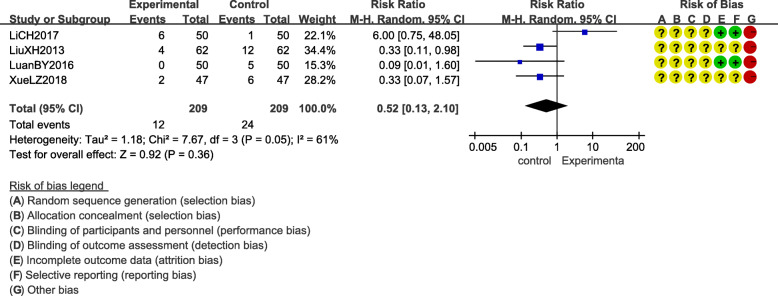
Adverse events of acupuncture compared to conventional conventional medicine

**Fig. 7:**
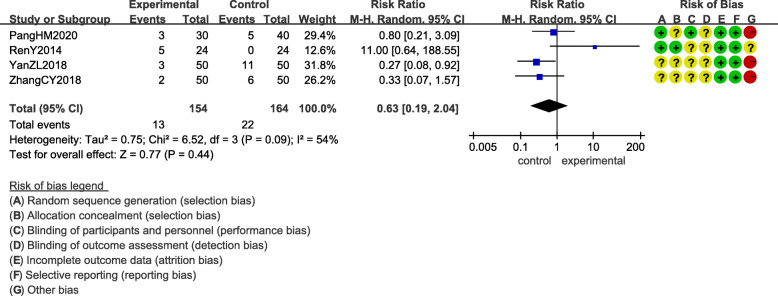
Adverse events of acupuncture combined with conventional conventional medicine compared to conventional conventional medicine

Adverse events were reported in eight out of the 13 included RCTs [13, 16, 18, 20–24] (Table 2). The differences in adverse events between acupuncture group and control group were shown in Figs. 6 and 7.

**Table 2:** Adverse events in included studies

Study ID	Sample size of adverse events	Intervention	Control
RenY2014	I: 5/24 C: 0/24	Acupuncture point bleeding	–
XueLZ2018	I:2/47 C:6/47	1 patient had nausea, 1 patient had dizziness	3 patients had nausea, 1 patient had vomiting, 2 patients had dizziness
LiuXH2013	I: 4/62 C: 12/62	2 patients had dizziness, 1 patient had vomiting, 1 patient had nausea	3 patients had dizziness, 6 patients had nausea, 3 patients had vomiting
LiCH2017	I: 6/50 C: 1/50	1 patients had dizziness	3 patients had nausea、1 patient had vomiting、2 patients had dizziness
ZhangZZ2018	I: 0/50 C: 5/50	–	1 patient had constipation,2 had mild gastrointestinal discomfort, 2 had spasmodic myalgia
YanZL2018	I: 3/50 C: 11/50	1 patient had nausea, 2 patients had dizziness	3 patients had nausea, 4 patients had vomiting, 4 patients had dizziness
ZhangCY2018	I: 2/50 C: 6/50	2 patients had dizziness	1 patients had nausea, 2 patients had vomiting, 3 patients had dizziness
PangHM2020	I: 3/30 C: 5/50	2 patient had vomiting, 1 patient had dizziness	3 patient had vomiting. 2 patient had dizziness

#### Publication bias

Six types of comparisons were involved in the 13 trials included. Each type of comparison involved no more than 10 trials, so inverted funnel plots were not appropriate to be conducted to evaluate publication bias.

## Discussion

### Summary of evidence

In this systematic review, the clinical effect and colonoscopy curative effect of acupuncture combined with mesalazine were better than that of mesalazine alone. The clinical effect of acupuncture alone was better than that of metronidazole combined with sulfasalazine. There was no significant difference in clinical effect between acupuncture alone and mesalazine alone. There were no significant differences in the adverse effects between acupuncture group and control group.

### Strengths and limitations

The diagnosis of UC lacks authoritative criteria and needs comprehensive analysis in many aspects. The latest consensus added “laboratory examination and imaging examination” to the previous criteria of diagnosing UC which was based on “clinical manifestations, endoscopic and histopathological manifestations”, emphasizing that the diagnosis of UC should combine objective tests and subjective descriptions to make comprehensive judgment [2]. Obviously, the studies included in this systematic review were too simple in the selection of outcome indicators and did not make a judgment on the effectiveness by integrating multiple factors.

In this systematic review, we conducted a systematic search and strictly assessed the original studies. Interventions were more restrictive than in the same type studies. However, the majority of the included studies had an unclear risk of bias in terms of random sequence generation and allocation concealment. Some of these studies also had an unclear risk of bias with regard to incomplete outcome data and selective reporting. In addition, the quality of evidence included in the studies was generally poor. Although it is undeniable that acupuncture may have potential effectiveness in treating UC, more high-quality trials are needed to prove it. Moreover, this systematic review did not limit the searching languages, but only retrieved Chinese and English databases, which may also increase the risk of bias. Therefore, we cannot draw firm conclusions based on the evidence of trials included in this review.

### Comparison with previous studies

We found two relative systematic reviews published in 2007 and in 2016 separately. Both reviews explored the effects of acupuncture mixed with moxibustion [26, 27]. In this review we prefer to clarify the effect of acupuncture first and therefore did not include studies of moxibustion, so as to provide more targeted evidence for clinicians when selecting acupuncture as the treatment for UC.

## Conclusions

Based on the evidence of this systematic review, we found that both acupuncture alone and acupuncture combined with conventional conventional medicine has a certain effect on the treatment of UC compared to conventional conventional medicine. Due to the limited number of clinical trials and generally poor methodological quality (according to the result of GRADE evidence profiles) of the included trials, high-quality randomized trials are needed to further validate the effectiveness and safety of acupuncture in the treatment of UC.

## Data Availability

All data analyzed in this study are supported by the published articles in databases, including six opening electronic databases (details in study identification and selection), and all data generated are included in this published article.

## Abbreviations

RCTs: Randomized clinical trials
UC: Ulcerative colitis
QOL: Quality of life
CNKI: China National Knowledge Infrastructure
VIP: Chinese Scientific Journal Database
CI: Confidence interval
MD: Mean difference
RR: Relative risk
I: Intervention
C: Control
NR: Not report
Y: Yes
N: No
